# Prion gene haplotypes of U.S. cattle

**DOI:** 10.1186/1471-2156-7-51

**Published:** 2006-11-08

**Authors:** Michael L Clawson, Michael P Heaton, John W Keele, Timothy PL Smith, Gregory P Harhay, William W Laegreid

**Affiliations:** 1 United States Department of Agriculture, Agricultural Research Service, U.S. Meat Animal Research Center (USMARC), Clay Center, NE 68933, USA

## Abstract

**Background:**

Bovine spongiform encephalopathy (BSE) is a fatal neurological disorder characterized by abnormal deposits of a protease-resistant isoform of the prion protein. Characterizing linkage disequilibrium (LD) and haplotype networks within the bovine prion gene (*PRNP*) is important for 1) testing rare or common *PRNP *variation for an association with BSE and 2) interpreting any association of *PRNP *alleles with BSE susceptibility. The objective of this study was to identify polymorphisms and haplotypes within *PRNP *from the promoter region through the 3'UTR in a diverse sample of U.S. cattle genomes.

**Results:**

A 25.2-kb genomic region containing *PRNP *was sequenced from 192 diverse U.S. beef and dairy cattle. Sequence analyses identified 388 total polymorphisms, of which 287 have not previously been reported. The polymorphism alleles define *PRNP *by regions of high and low LD. High LD is present between alleles in the promoter region through exon 2 (6.7 kb). *PRNP *alleles within the majority of intron 2, the entire coding sequence and the untranslated region of exon 3 are in low LD (18.0 kb). Two haplotype networks, one representing the region of high LD and the other the region of low LD yielded nineteen different combinations that represent haplotypes spanning *PRNP*. The haplotype combinations are tagged by 19 polymorphisms (htSNPS) which characterize variation within and across *PRNP*.

**Conclusion:**

The number of polymorphisms in the prion gene region of U.S. cattle is nearly four times greater than previously described. These polymorphisms define *PRNP *haplotypes that may influence BSE susceptibility in cattle.

## Background

Transmissible spongiform encephalopathies (TSEs) have been identified in humans, sheep, goats, deer, elk, moose, cattle, cats, and mink [1]. A cattle TSE, bovine spongiform encephalopathy (BSE), was first diagnosed among Holstein/Friesian cattle in the United Kingdom [2] and has since been detected in at least twenty five countries including the United States. The BSE agent is the probable cause of the human TSE, variant Creutzfeldt-Jakob Disease (vCJD) [3,4], transmitted from cattle to people via the food chain.

Variation in the prion gene (*PRNP*) correlates with TSE progression in humans [5,6], sheep [7], and mice [8]. In cattle, a 23-bp insertion/deletion (indel) polymorphism in the putative promoter region and a 12-bp indel within intron I have been associated with German BSE-affected animals [9]. These polymorphisms are present in U.S. cattle [10]. However, most of *PRNP *has not been characterized in a population as diverse as U.S. cattle outside of the coding region and 3'UTR of exon III, and portions of the promoter and intron I [10-12]. Consequently, the extent of *PRNP *polymorphisms, linkage between *PRNP *alleles, recombination events, and haplotype diversity within *PRNP *is not known.

Public health concerns associated with vCJD and economic impacts of BSE on the cattle industry worldwide compel a thorough characterization of the genetic variation of bovine *PRNP*. Single nucleotide polymorphism (SNP) discovery in small populations introduce ascertainment biases of SNP properties [13,14], and partial sequencing of genes in deep populations may characterize haplotype networks that extend past the sequenced region [15], yet still miss significant variation within the gene. The aim of this study was to characterize the extent of linkage disequilibrium (LD) and haplotype networks within *PRNP *ranging from the promoter past the 3'UTR (25.2 kb) in 192 U.S. cattle (16 beef and five dairy breeds). Reported here are 287 newly identified *PRNP *polymorphisms, the frequencies of 388 *PRNP *polymorphisms in U.S. beef and dairy cattle, a reference map of LD and haplotypes throughout *PRNP*, and the identification of 19 haplotype tagging SNPs (htSNPs) that are effective in U.S. populations of cattle. These results provide a reference framework for accurate and comprehensive evaluation of *PRNP *variation and its relationship to BSE.

## Results

### Amplification and sequence coverage of the bovine *PRNP *gene from the promoter region through the 3'UTR (25.2 kb) in U.S. cattle

**Figure 1:**
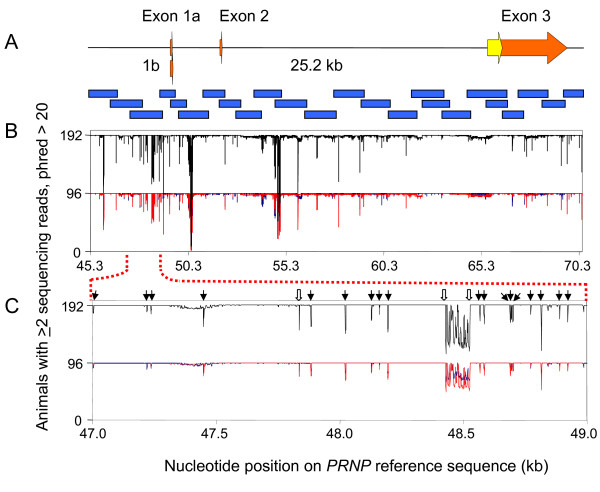
**Sequence coverage of *PRNP *in U.S. beef and dairy cattle**. **A**. Physical map of *PRNP *that was sequenced in 192 beef and dairy cattle (25.2 kb) on 24 overlapping amplicons (blue bars). Orange and yellow color arrows represent untranslated and coding regions, respectively. **B**. Overall quality of *PRNP *sequence coverage. *PRNP *nucleotide sequence. with a phred score greater than 20 from at least two sequencing reads from the same animal was mapped to the corresponding nucleotide on a reference sequence [GenBank:AJ298878]. The blue line depicts the sequence quality of the 192 haploid cattle genomes comprising MBCDP2.1, the red line represents the 192 haploid cattle genomes comprising MDCP1.5, and the black line represents all 384 genomes combined. **C**. Close-up of *PRNP *sequence quality in an arbitrarily selected 2-kb window. Vertical arrows represent the location of polymorphisms (black for SNPs and white for indels). Polymorphisms with high minor allele frequencies are reflected by dips in sequence quality that correspond with heterozygous animals. The low phred score locus approximately at 48.5 kb is the result of animals heterozygous for two indels.

The *PRNP *gene was sequenced in 192 beef and dairy cattle; 16 beef and five dairy breeds from 24 overlapping amplicons (Figure 1 and see additional file 1). The amplification primers do not hybridize with *PRNP *regions containing any of the polymorphisms observed in this study, nor do any of the 150 sequencing primers used for redundant coverage of *PRNP *nucleotides (see additional file 1). Two or more high quality or unambiguous heterozygous reads were obtained for each *PRNP *nucleotide throughout 24.8 kb of the 25.2 kb region for approximately 95% of the cattle. Regions of *PRNP *that correspond with ambiguous sequence from more than five percent of the 192 animals were identified in the promoter region (95 bp), intron 1 (96 bp), and intron 2 (225 bp). These regions are attributable to closely positioned indels with heterozygous genotypes of high frequency in the cattle populations or stretches of mononucleotide repeats, both of which interfere with collection of high quality sequence. The positioning of these problematic loci was such that it was not possible to design amplification/sequencing reactions to cover the areas with high quality sequence.

### *PRNP *polymorphisms in U.S. cattle

**Figure 2:**
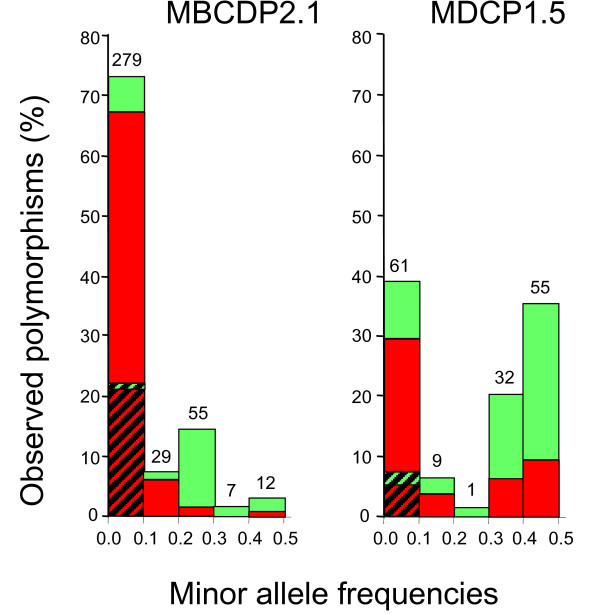
***PRNP *polymorphism minor allele frequencies in U.S. beef (MBCDP2.1, n = 96) and dairy cattle (MDCP1.5, n = 96)**. Hatched bars represent the proportion of polymorphisms where the minor allele was observed in only one animal (singleton). Green and red colors represent the proportion of polymorphisms that have or have not been reported, respectively. Polymorphism numbers are show above bars. Multi-allelic polymorphisms are not represented.

**Figure 3:**
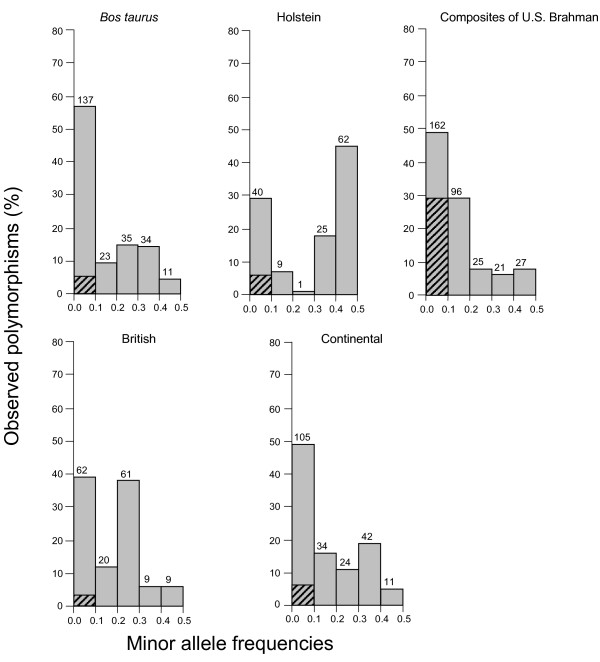
***PRNP *polymorphism frequencies in U.S. cattle populations**. *PRNP *polymorphism frequencies are shown for populations of *Bos taurus *(n = 94), Holstein (n = 86), Composite of U.S. Brahman (n = 20), British (n = 39), and Continental (n = 51). Hatched bars represent the proportion of polymorphisms where the minor allele was observed in only one animal (singleton). Polymorphism numbers are shown above the bars.

A total of 388 polymorphisms (351 SNPs and 37 indels) were observed in *PRNP *gene sequences from the 384 chromosomes present in all 192 cattle (Figure 2 and see additional file 2). Two hundred and eighty-seven of the polymorphisms were not described in GenBank and literature searches as of July 10, 2006, and all were identified in non-coding regions of *PRNP*. The majority of polymorphisms (382/388) were observed in the multi-breed beef diversity panel (17 breeds, 192 chromosomes). In contrast, 158 polymorphisms were observed in the multi-breed dairy diversity panel (five breeds, 192 chromosomes), of which six were unique to the panel. Polymorphisms were observed in subgroups of cattle as follows (Figure 3): *Bos taurus *(240/388), British (161/388), Continental (216/388), Composites of U.S. Brahman (331/388), and Holstein (137/388). A total of 261 polymorphisms were used for haplotype inference across the five subgroups of cattle. One hundred and twenty polymorphisms were excluded from haplotype inference in all five subgroups of cattle due to low minor allele frequencies and an additional seven were excluded by Hardy-Weinberg testing. Sixty three of the 388 polymorphisms were only observed in one animal of the multi-breed beef diversity panel, a composite of U.S. Brahman.

### Linkage disequilibrium of *PRNP *alleles

**Figure 4:**
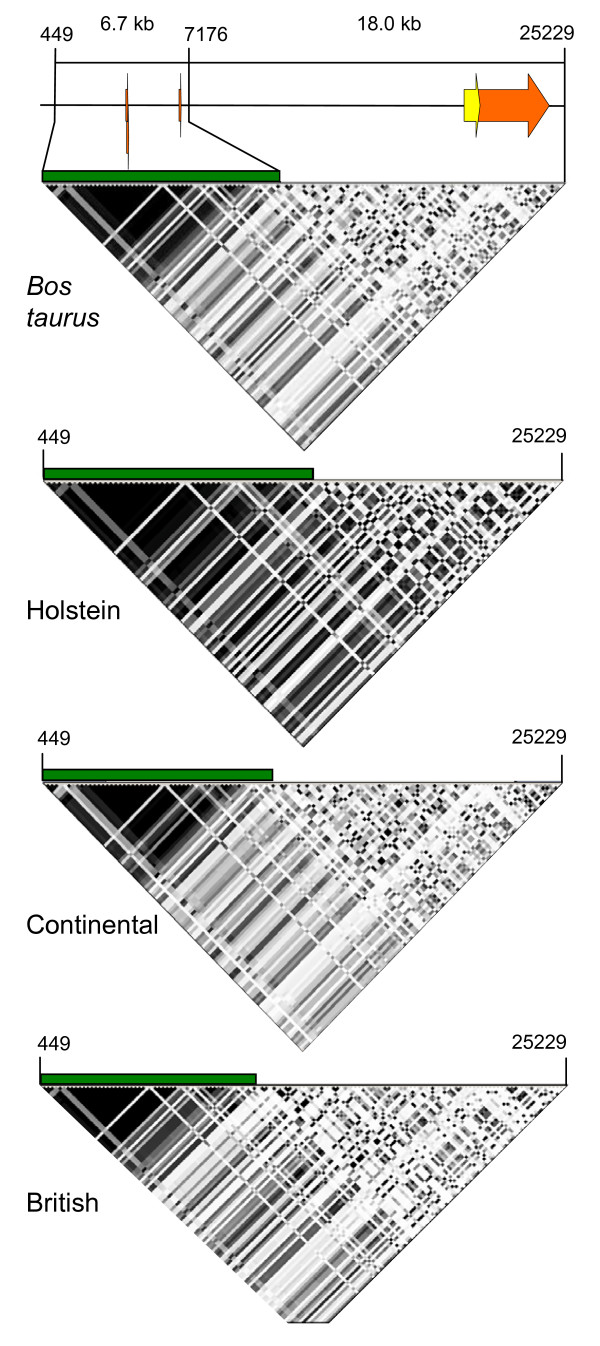
**Pairwise plots of LD between *PRNP *alleles in U.S. cattle populations**. Numbers refer to the physical location of polymorphisms in a *PRNP *consensus sequence file [GenBank:DQ457195] and the LD plots. Dark and light shading indicate high and low r^2 ^values, respectively. Horizontal green bars highlight the region of high LD within *PRNP *that is flanked by polymorphisms at positions 449 and 7176.

A 6.7-kb region of high LD was identified in U.S. beef and dairy cattle from the 5' promoter region through exon 1, intron 1, exon 2, and part of intron 2 (Figure 4). Adjacent to the high LD region, an 18.0-kb portion of *PRNP*, containing the majority of intron 2 and all of exon 3, displayed markedly less LD (region of low LD). The region of high LD was not restricted to a cattle subgroup and was observed in the beef cattle diversity panel; dairy cattle diversity panel, *B. taurus*, British, Continental, and Holstein cattle subgroups (minor allele frequency ≥ 0.05, Hardy Weinberg p > 0.01). Including the polymorphisms with minor alleles of low frequency, 115 polymorphisms were identified in the 6.7 region of high LD, of which 45 have alleles in LD.

### Haplotype inference and networks

**Figure 5:**
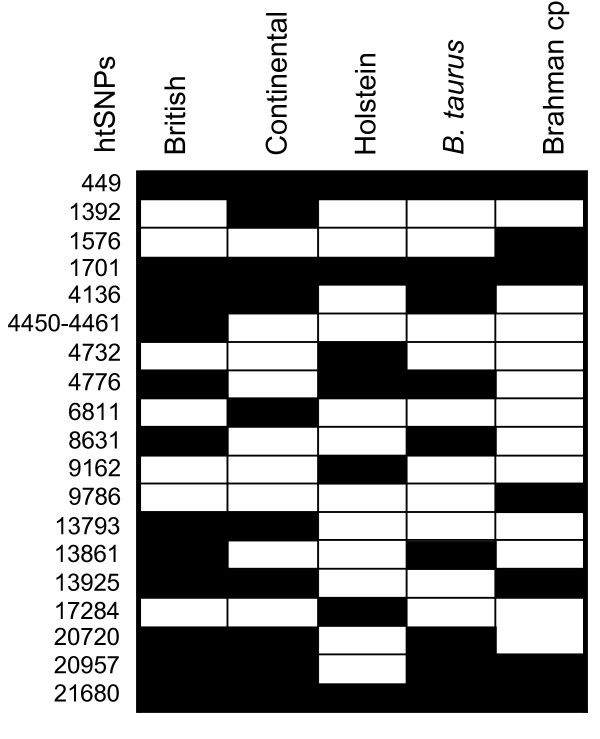
**Haplotype tagging SNPs in five U.S. cattle populations**. Numbers indicate the nucleotide position of htSNP alleles in a *PRNP *consensus sequence file [GenBank:DQ457195]. Black boxes indicate the cattle populations in which an SNP defines a haplotype.

**Figure 6:**
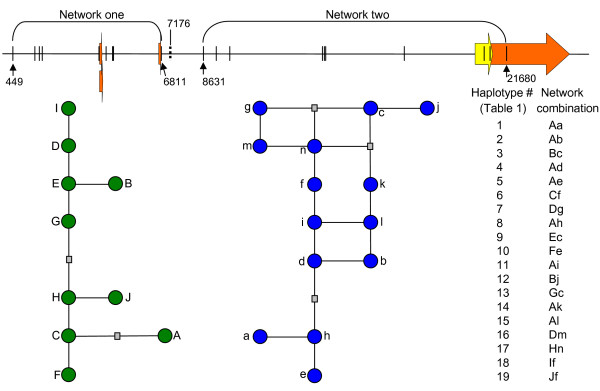
**Bovine *PRNP *haplotypes defined by two Median-Joining networks**. Two networks were constructed from 19 htSNPs. Network one was generated from htSNPs 449–6811 (n = 9), and network two from htSNPs 8631–21680 (n = 10). The physical positions of all 19 htSNPs are shown with solid vertical lines along the *PRNP *physical map. The dashed vertical line shows the position of a non-htSNP at nucleotide 7176 of the *PRNP *consensus sequence file [GenBank:DQ457195]. Circles represent extant *PRNP *haplotypes and are not scaled to frequency. Grey rectangles represent *PRNP *haplotypes not observed in our sample. Sequence for each network combination is presented in Table 1.

Nineteen *PRNP *haplotypes were inferred by the Expected Maximization (EM) algorithm on at least four chromosomes in one or more of the following subgroups: *B. taurus*, British, Continental, Holstein, and U.S. Brahman composite. Six of the haplotypes were observed in a homozygous state in one or more cattle subgroup. The 19 *PRNP *haplotypes represented 62% or more of the haploid genomes in all subgroups of cattle except U.S. Brahman composite (42%) and could be defined by the alleles of 19 htSNPs (Figure 5, Table 1). Because the haplotypes span two distinct *PRNP *regions defined by high and low LD, a Median-Joining network for each region was constructed (Figure 6). The network within the 6.7-kb region of high LD contains "sub-haplotypes" phased from nine of the 19 htSNPs and shows a linear stepwise relationship of alleles (Figure 6; network 1, Table 1). The network within the *PRNP *region of low LD contains sub-haplotypes phased from the remaining ten htSNPs and has a distinctly looped structure, indicating multiple unresolved allele relationships (Figure 6; network 2, Table 1). Sub-haplotype combinations from the two networks effectively account for the regions of high and low LD and yield haplotypes that span *PRNP *(Figure 6).

**Table 1:** Bovine *PRNP *haplotype frequencies in populations of U.S. cattle

	Network #1^a^									Network #2										Haplotype frequencies				
			
Haplotype																				n = 188^d^	n = 172	n = 78	n = 102	n = 40
																								
#	449^b^	1392	1576	1701	4136	4450–4461^c^	4732	4776	6811	8631	9162	9786	13793	13861	13925	17284	20720	20957	21680	*Bos taurus*	Holstein	British	Continental	Indicus cp^e^
1	T	C	C	G	C	Z	G	C	A	A	T	T	G	C	G	G	T	C	T	0.102	-	0.158	0.067	0.125
2	T	C	C	G	C	Z	G	C	A	A	T	T	G	C	C	G	C	C	C	0.100	0.264	0.083	0.099	0.075
3	G	C	C	A	C	I	A	T	A	G	T	C	A	G	C	G	C	C	C	0.087	0.059	0.044	0.088	-
4	T	C	C	G	C	Z	G	C	A	A	T	T	G	C	C	G	C	C	T	0.081	-	0.075	0.073	0.075
5	T	C	C	G	C	Z	G	C	A	A	T	T	G	C	G	G	C	T	T	0.071	0.030	0.079	0.029	-
6	T	C	C	A	C	Z	G	C	A	G	T	T	A	C	C	G	C	C	T	0.049	-	0.026	0.078	0.025
7	G	C	C	A	T	I	A	C	A	G	C	C	A	G	C	G	C	C	T	0.033	-	0.039	-	-
8	T	C	C	G	C	Z	G	C	A	A	T	T	G	C	G	G	C	C	T	0.027	-	-	0.069	-
9	G	C	C	A	C	I	A	C	A	G	T	C	A	G	C	G	C	C	C	0.027	0.042	0.074	-	-
10	T	C	C	A	C	Z	G	C	T	A	T	T	G	C	G	G	C	T	T	0.027	-	0.026	0.029	-
11	T	C	C	G	C	Z	G	C	A	A	T	T	A	C	C	G	C	C	T	0.024	-	-	0.029	-
12	G	C	C	A	C	I	A	T	A	G	T	C	A	G	C	A	C	C	C	- f	0.091	-	-	-
13	G	C	C	A	C	I	G	C	A	G	T	C	A	G	C	G	C	C	C	-	0.084	-	-	-
14	T	C	C	G	C	Z	G	C	A	A	T	T	A	G	C	G	C	C	C	-	0.053	0.036	-	-
15	T	C	C	G	C	Z	G	C	A	A	T	T	A	C	C	G	C	C	C	-	0.052	-	-	-
16	G	C	C	A	T	I	A	C	A	G	C	T	A	G	C	G	C	C	T	-	0.032	-	0.029	-
17	T	C	C	A	C	I	G	C	A	G	T	T	A	G	C	G	C	C	T	-	0.030	-	-	-
18	G	T	C	A	T	I	A	C	A	G	T	T	A	C	C	G	C	C	T	-	-	-	0.039	-
19	T	C	T	A	C	I	G	C	A	G	T	T	A	C	C	G	C	C	T	-	-	-	-	0.123
Total haplotype frequency																				0.628	0.737	0.640	0.629	0.423

^a^Networks 1 and 2 are shown in figure 5.

^b^Nucleotide position in GenBank accession # DQ457195.

^c^Twelve base InDel, I = GGGGGCCGCGGC, Z = deletion.

^d^Number of haploid genomes.

^e^Composites of U.S. Brahman.

^f^Indicates 0.000 frequency in population.

## Discussion

Sequencing the *PRNP *gene in 192 cattle representing 21 breeds resulted in the identification of 388 polymorphisms and detection of a region of high LD in the 5' non-coding region of *PRNP*. We identified 19 common *PRNP *haplotypes in U.S. cattle and characterized 19 htSNPs with the power to monitor these haplotypes. These results provide the means and a context for testing *PRNP *variation for an association with BSE.

Cattle present a challenge for LD analysis due to their complex history. Current haplotype patterns in domestic cattle are influenced by an evolutionary split of *B. taurus *and *Bos indicus *subspecies over 100,000 years ago [16], stratification of *B. taurus *germplasm within British and Continental populations, multiple population bottlenecks, gene migration, and inter-subspecies breeding. The cattle samples used in this study were classified into subgroups that addressed their natural history, an approach applied in other studies [15,17]. Analyses of cattle subgroups by *B. taurus*, British, Continental, and Holstein classifications yielded similar LD regions within *PRNP*, indicating that the LD is consistent within and between *B. taurus *populations. A similar trend was observed within the small sample of *B. indicus *germplasm that was represented by the U.S. Brahman composite subgroup. The conservation of LD in *PRNP *indicates that some htSNPs should apply across populations.

Two regions of *PRNP *reflect different population histories (recombination, gene conversion, allelic fixation) by both pairwise r^2 ^measurements of LD and haplotype Median-Joining Networks. Pairwise r^2 ^values are lowered by historical recombination and the emergence of alleles on different haplotype lineages [17]. Consequently, alleles with high pairwise r^2 ^values predict linear haplotype networks, and alleles with low pairwise r^2 ^values predict complex or looped haplotype networks. The region of high LD that encompasses a 6.7-kb portion of *PRNP *extends to the 5' boundary of the *PRNP *locus sequenced in this study. Additional high LD may extend upstream of the sequenced locus. *PRNP *SNP alleles within this region are highly correlated with each other, and haplotypes phased within the region yield a linear network. In contrast, 18.0 kb of *PRNP*, which includes the entire protein coding and untranslated region of exon 3, has alleles in low LD and the associated haplotype network is complex. Alleles in this region may not be correlated with alleles elsewhere on the chromosome.

A previous study implicated a 23-bp indel in the promoter region and a 12-bp indel within intron 1 of *PRNP *with an association with susceptibility to BSE [9]. Both of these polymorphisms lie within the region of high LD described here, and their alleles are strongly correlated with the alleles of 43 other polymorphisms detected in this study. Until both chromosomal boundaries flanking the region of LD are determined, the number of polymorphisms with alleles in LD with those associated with BSE is unknown.

Although the diversity panels used to sequence *PRNP *represent a broad sample of U.S. cattle, it is likely that additional diversity within *PRNP *is present at low frequency in U.S. herds. This hypothesis is supported by the *PRNP *sequence from a single animal that accounted for 16.2 % of all observed polymorphisms. Some countries, including the U.S., have detected atypical BSE cases at exceedingly low frequencies with increased surveillance [18-20]. Atypical BSE can differ from typical BSE by brain distribution and plaque morphology of the protease-resistant prion isoform (PrP^res^), or by western immunoblot profile of PrP^res ^following proteinase K digestion [18-20]. Diverse chromosomes in cattle populations could confound interpretations of *PRNP *variation identified from individual cases of either typical or atypical BSE.

## Conclusion

The number of polymorphisms in the prion gene region of U.S. cattle is nearly four times greater than previously described. *PRNP *is divided into regions of high and low LD. The 19 htSNPs identified in this study define haplotype combinations from the two *PRNP *regions that may influence BSE susceptibility in cattle.

## Methods

### DNA panels used for *PRNP *DNA polymorphism discovery

The U.S. Meat Animal Research Center (USMARC) Beef Cattle Discovery Panel 2.1 (MBCDP2.1) was used to sample the breadth of *PRNP *genetic diversity in popular U.S. beef breeds. The selection and assembly of this panel has been previously described [21]. MBCDP2.1 consists of 96 bulls from the following breeds; Angus (n = 8), Hereford (n = 8), Limousin (n = 8), Simmental (n = 7), Charolais (n = 6), Beefmaster (n = 5), Red Angus (n = 6), Gelbvieh (n = 6), Brangus (n = 5), Salers (n = 5), Brahman (n = 6), Shorthorn (n = 5), Maine-Anjou (n = 5), Longhorn (n = 4), St. Gertrudis (n = 4), Chianina (n = 4), and Holstein (n = 4). The USMARC Dairy Cattle Panel (MDCP1.5) was used to sample the breadth of *PRNP *genetic diversity in dairy cattle and was a dairy subset of the USMARC-FSIS Random Market Cattle Panel version 1.1 described elsewhere [22]. MDCP1.5 consists of 96 dairy cows of the following breeds; Holstein (n = 82), Jersey (n = 7), Guernsey (n = 3), Aryshire (n = 2), and Brown Swiss (n = 2).

### Primer design, PCR, and cycle sequencing

Reference sequence for the bovine *PRNP *gene was used as a template for primer design [GenBank:AJ298878], [23,24]. Primers were designed to amplify 24 overlapping amplicons that collectively span a 25.2-kb region of *PRNP *(Oligo 6.61). Nested sequencing primers were designed for each amplicon to provide nucleotide coverage in both directions. Following preliminary experiments of amplification primer performance, the 192 cattle genomes comprising MBCDP2.1 and MDCP1.5 were subjected to 40 rounds of PCR with conditions as described [25] (see additional file 1). Following an Exonuclease I digestion [26], the *PRNP *amplicons were sequenced with BigDye terminator chemistry on an ABI 3730 capillary sequencer (PE Applied Biosystems, Foster City, CA).

### Polymorphism detection, sequence quality, and coverage

Sequences from the 192 animals of the multi-breed beef and dairy panels were processed for polymorphism detection with Phred and Phrap [27,28], Polyphred 3.5 [29], and Consed software [30]. A physical map linked to the *PRNP *consensus sequence and the location of polymorphisms was constructed in Vector NTI (v7.1). The map was annotated with all amplification and sequencing primers connected with the *PRNP *sequence. Replacement primers for those that hybridized to genomic loci containing polymorphisms were designed and used for additional amplification and sequencing of *PRNP *regions. *PRNP *nucleotide sequence with a phred score greater than 20 from at least two sequencing reads from the same animal was mapped to the corresponding nucleotide on reference sequence [GenBank:AJ298878]. Sequence compromised by SNP loci under associated amplification or sequencing primers was not analyzed for sequence coverage or the determination of genotypes. Regions reflecting poor sequence quality (<95% animal coverage) were identified, additional amplicons and sequencing primers were designed, and additional sequencing was performed. *PRNP *allele genotypes were mapped to reference sequence [GenBank:AJ298878] and stored in a relational database. A file of *PRNP *sequence annotated with all polymorphisms observed in this study and their frequencies in the beef diversity panel (MBCDP2.1) and dairy diversity panel (MDCP1.5) has been deposited in GenBank [GenBank:DQ457195].

### Definitions of animal subgroups

The 192 beef and dairy animals whose genomic DNA comprise diversity panels MBCDP2.1 and MDCP1.5 were sorted into five subgroups based on breed composition. The *B. taurus *subgroup (n = 94) consisted of Angus (n = 8), Hereford (n = 8), Limousin (n = 8), Simmental (n = 7), Charolais (n = 6), Red Angus (n = 6), Gelbvieh (n = 6), Salers (n = 5), Shorthorn (n = 5), Maine-Anjou (n = 5), Texas Longhorn (n = 4), Chianina (n = 4), Aryshire (n = 2), Brown Swiss (n = 2), Guernsey (n = 3), Jersey (n = 7), and Holstein (n = 8; randomly selected from 86 to avoid over-representation of the Holstein breed). The British subgroup (n = 39) consisted of Angus (n = 8), Hereford (n = 8), Red Angus (n = 6), Shorthorn (n = 5), Aryshire (n = 2), Jersey (n = 7), and Guernsey (n = 3). The Continental subgroup (n = 51) consisted of Charolais (n = 6), Chianina (n = 4), Gelbvieh (n = 6), Limousin (n = 8), Maine-Anjou (n = 5), Salers (n = 5), Simmental (n = 7), Brown Swiss (n = 2), and Holstein (n = 8; randomly selected from 86). The Holstein subgroup consisted of 86 Holsteins. The final subgroup, Composites of U.S. Brahman (n = 20) consisted of Beefmaster (n = 5), Brahman (n = 6), Brangus (n = 5), and Santa Gertrudis (n = 4).

### LD estimation, haplotype inference, and median-joining network analyses

Unphased *PRNP *genotypes were assembled for each animal in datasets of the *B. taurus*, British, Continental, Holstein, and Composite of U.S. Brahman subgroups. Polymorphisms with more than two alleles, a minor allele frequency <0.05, or those not in Hardy-Weinberg equilibrium (Chi-square p < 0.01) were excluded from further analyses. Our cattle populations were not the result of random mating, violating an assumption of Hardy-Weinberg equilibrium. However, the Hardy-Weinberg test facilitated the identification of common haplotypes within the subgroups by excluding polymorphisms where the minor allele was amplified in a particular breed, yet had a low overall frequency.

The extent of LD between the *PRNP *alleles of each dataset was calculated with pairwise r^2 ^values (Haploview v3.2 [31]). Regions of LD were determined through visual inspection of LD graphs. Haplotypes were inferred in Haploview using the EM algorithm and a minimal set of polymorphisms was identified that collectively tagged all observed haplotypes predicted on four or more chromosomes in one or more of the five subgroups. The htSNPs identified across the five subgroup datasets were combined into a single set of 19 htSNPS. The 19 htSNPs were used to infer haplotypes within the five subgroup datasets. Median-Joining networks of *PRNP *haplotypes were constructed in Network (v4.111)[32].

## Authors' contributions

MLC participated in the project conception and design, data generation and management, sequence analyses, polymorphism genotyping, LD and haplotype analyses, and drafted the manuscript. MPH participated in the project conception and design, data generation, sequence analyses, script development regarding sequence coverage and polymorphism genotype mapping, and suggested improvements regarding the data pipeline. JWK participated in sequence analyses and wrote scripts for a semi-automated pipeline that stored DNA trace files and polymorphism genotypes within a relational database and mapped sequence phred scores and polymorphism genotypes onto a reference sequence. TPLS performed *PRNP *sequencing, analyzed sequence, and suggested improvements regarding the data pipeline. GPH participated in LD and haplotype analyses. WWL participated in the project conception and design, data generation, LD and haplotype analyses, and suggested improvements regarding the data pipeline.

## Supplementary Material

Additional file 1 **Oligonucleotides for *PRNP *amplification and sequencing**. Annealing location, USMARC number, DNA sequence, orientation, function, and annealing temperature for 198 oligonucleotides used for *PRNP *amplification and sequencing. Click here for file

Additional file 2 **Allele and genotype frequencies of *PRNP *polymorphisms in the USMARC beef and dairy cattle panels**. Physical positions and *PRNP *regions of all 388 polymorphisms observed in this study; their allele and genotype frequencies in the beef and dairy cattle panels, and the GenBank accession numbers for 101 polymorphisms previously reported. Click here for file
